# Heavy metals detected in fetal and placental tissues in a pregnancy complicated by severe fetal growth impairment and sacrococcygeal teratoma: a case report

**DOI:** 10.3389/ftox.2026.1722241

**Published:** 2026-03-19

**Authors:** Riccardo Addobbati, Giuliano Pesel, Donatella Sansone, Francesca Moltrasio, Giovanna Ricci, Tommaso Livieri, Martina Franzin, Paolo Bailo

**Affiliations:** 1 Institute for Maternal and Child Health, IRCCS “Burlo Garofolo”, Trieste, Italy; 2 Section of Legal Medicine, School of Law, University of Camerino, Camerino, Italy; 3 School of Specialization in Occupational Medicine, University of Trieste, Trieste, Italy; 4 Sc Anatomic Pathology ASST Brianza- Presidio Pio XI, Desio, Italy; 5 Department of Medical, Surgical and Health Sciences, University of Trieste, Trieste, Italy

**Keywords:** fetal malformations, occupational exposure, paraffin-embedded tissue analysis, pregnancy and toxicants, sacrococcygeal teratoma

## Abstract

Protecting the health of pregnant workers is a critical public health and occupational priority. This case report describes a textile industry worker with long-term occupational chemical exposure whose pregnancy was complicated by severe fetal malformations leading to termination; a subsequent multidisciplinary reassessment supported an occupationally mediated contributory role. The potential environmental/occupational contribution was not recognised during the initial routine clinical work-up and was identified only retrospectively through integrated toxicology, pathology, occupational medicine, and forensic evaluation. A distinctive aspect of the case was the application of an innovative technique to detect toxicants in formalin-fixed, paraffin-embedded (FFPE) tissue samples years after the initial events, expanding the possibilities for retrospective environmental exposure assessment. Additionally, the co-occurrence of a sacrococcygeal teratoma (SCT) is discussed as a hypothesis-generating observation in the context of possible occupational exposure. This case underscores the importance of comprehensive occupational health surveillance during pregnancy, the integration of environmental exposure assessment into prenatal care, and highlights the potential regulatory and occupational-health implications of inadequate protection of pregnant workers.

## 1 Introduction

Occupational diseases continue to represent a critical public health concern, particularly when they affect not only workers themselves but also their unborn children. Among the various sectors where occupational hazards are prevalent, the textile industry stands out for its extensive use of chemical agents—such as solvents, dyes, and finishing agents—which may have deleterious effects on reproductive health (Wong et al., 2009; Duong et al., 2011). Female workers of reproductive age, especially those who are pregnant or planning to conceive, face increased vulnerability to these toxic exposures. These exposures may result in a range of adverse reproductive outcomes, including infertility, miscarriage, preterm birth, low birth weight, and congenital anomalies (Khattak et al., 1999; Spinder et al., 2019; Kumar et al., 2019). Despite growing awareness of occupational reproductive risks, pregnancy-related conditions remain notoriously underdiagnosed in this context. This is due in part to the complex and multifactorial nature of such conditions, which can be influenced by both occupational and non-occupational factors (Figà-Talamanca, 2006; Baranski, 1993). Moreover, the latent or delayed manifestation of adverse outcomes complicates the process of establishing a causal link between occupational exposure and reproductive harm (Wigle et al., 2008; Zhang et al., 1992; Desrosiers et al., 2012). As a result, these conditions often go unrecognized within workplace health surveillance systems, and affected workers may experience delays or barriers in recognition within surveillance and compensation pathways (Flynn et al., 2015; Sexton et al., 1993). To address these challenges, a multidisciplinary approach is essential—one that integrates forensic medicine, occupational health, and toxicology. Forensic expertise is particularly valuable in evaluating the plausibility of occupational etiology in reproductive disorders and in documenting the evidence necessary for legal and compensation claims (Mazanti et al., 2020). At the same time, occupational health professionals play a critical role in identifying hazardous exposures, implementing preventive measures, and advocating for safer workplace environments, especially for pregnant workers (Jurewicz and Hanke, 2011; Hanke and Jurewicz, 2004).

## 2 Timeline

**Table:** 

Date	Event
January 2002	Start of employment
Spring 2002	Onset of dermatopathy symptoms
20 December 2016	Last menstrual period (beginning of pregnancy)
24 January 2017	Commencement of work abstention
15 May 2017	Termination of pregnancy

## 3 Narrative

The patient, a female textile industry worker, commenced employment at 18 years of age and remained employed in the sector until 2021, with duties spanning weaving-related activities, textile sample preparation, and fabric quality control. From early adulthood, she developed occupational dermatoses consistent with allergic contact dermatitis, primarily attributed to nickel sulfate exposure, accompanied by allergic conjunctivitis. Eczematous eruptions affecting the hands and forearms showed a positive stop–restart (removal-from-exposure) pattern. These cutaneous manifestations were persistent throughout her professional activity, with partial remission noted only during maternity leave.

In 2008, epicutaneous (patch) testing revealed sensitisation to nickel sulfate and potassium dichromate. Subsequent testing in the following years demonstrated additional sensitisation to hydroxyethyl methacrylate, formaldehyde, and benzalkonium chloride.

The maternal family history was negative for congenital malformations. Behavioural and lifestyle factors were assessed. The patient reported minimal e-cigarette use (<1/day) and denied alcohol consumption, illicit drug use, and medications or non-prescribed supplements; she had been taking standard preconception/pregnancy supplementation (including folic acid) for at least 1 year as prescribed. Diet was reported as balanced without high-risk dietary exposures (caffeine ∼ two coffees/day), and no relevant domestic or hobby-related chemical exposures were identified; she adhered to recommended prenatal care with regular primary-care access and scheduled obstetric follow-up. Sleep was reported as regular, with only modest work-related stressors.

On 24 January 2017, at 33 years old, the patient ceased occupational activity due to pregnancy (last menstrual period: 20 December 2016; gestational age: 5 weeks). Laboratory investigations dated 26 January 2017 showed Rubella and Cytomegalovirus IgG positivity with IgM negativity, consistent with pre-existing immunity/prior exposure and without serological evidence of recent primary infection; tests were negative for Toxoplasma gondii, *Treponema pallidum* (TPHA, VDRL), and HIV.

**FIGURE 1:**
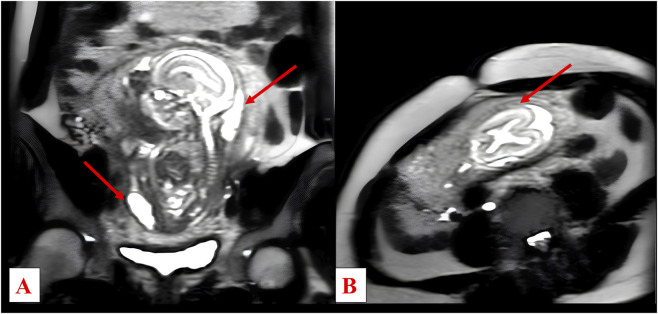
Low-resolution T2-weighted MRI scans. **(A)** Sagittal plane of the fetus showing oligohydramnios/anhydramnios (arrows). **(B)** Coronal plane of the fetal head showing a slightly reduced biparietal diameter and a morphological tendency toward scaphocephaly, with the cranial contour highlighted by an arrow.

Ultrasound performed on 6 April 2017 detected oligohydramnios with non-visualisation of the renal fossae. A subsequent scan confirmed the oligohydramnios and raised suspicion of bilateral renal malformation. Amniocentesis was conducted for fetal karyotyping and chromosomal microarray (array-CGH), and intra-amniotic transfusion was performed. A detailed anatomical ultrasound on 13 April 2017 revealed dolichocephaly, ventriculomegaly, and a suspected “lemon sign.” Additional findings included a sacrococcygeal mass consistent with teratoma, dysmorphic sacral/coccygeal spine, and a cardiac anomaly requiring further evaluation. The estimated fetal weight was 146 g. Cytogenetic analyses revealed a normal male karyotype with no pathogenic copy number variants; fetal chromosomal microarray yielded no result due to insufficient material. A follow-up ultrasound on 8 May 2017 demonstrated progression to anhydramnios and bilateral cystic renal dysplasia. Fetal MRI confirmed severe oligohydramnios/anhydramnios and a 20 mm, irregular cystic lesion in the anterolateral pelvic region at the sacrococcygeal level, suggestive of bladder exstrophy. Cranial biometry showed anteroposterior predominance at the upper limit of normal and a tendency toward scaphocephaly. The brain parenchyma appeared mildly immature relative to gestational age (Figure 1). At 21 weeks’ gestation, termination of pregnancy was carried out via pharmacological induction. Postmortem examination of the fetus revealed a normally developed male with a double sacrococcygeal teratoma and phocomelia of the right upper limb. The placenta exhibited chorionic villi consistent with the second trimester, and the umbilical cord was noted to be two-vessel (bivascular). Following the event, the couple underwent post-termination genetic counselling and parental genetic investigations to inform recurrence risk in future pregnancies; no clinically significant abnormalities were identified.

On retrospective review, some findings appeared to have been under-appreciated in the initial postmortem report. It was not until 8 years later that the case was referred to our team for a comprehensive aetiological reassessment, with particular attention to whether occupational exposures could reasonably be considered a likely contributory factor in severe fetal malformations that led to termination of pregnancy.

In addition to the available clinical and instrumental documentation, the only biological material retained for further investigation consisted of formaldehyde-fixed, paraffin-embedded (FFPE) tissue samples obtained during the original postmortem examination. To explore the potential occupational aetiology of the fetal pathology, a multidisciplinary investigative approach was employed. This included detailed histopathological and toxicological analyses of the preserved tissues, as well as an occupational medicine evaluation to reconstruct the nature and extent of the patient’s exposure to specific chemical agents in the workplace.

Histopathological examination was conducted on FFPE sections stained with haematoxylin and eosin. The tissues selected for histopathological evaluation included the placenta, fetal kidneys, and the sacrococcygeal teratoma (SCT).

**FIGURE 2:**
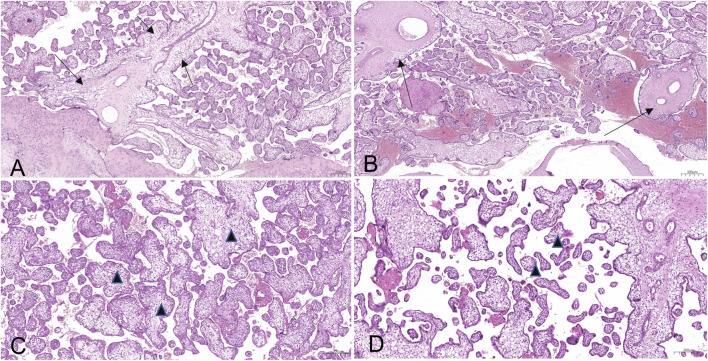
Sections of placental parenchyma, stained with hematoxylin and eosin (EE). Evident hydropic and edematous vacuolation of the chorionic villi in the different stages of branching. Vacuoles affecting the villi anchoring to the maternal decidual plate (**A**, arrows), the stem villi (**B**, arrows), and the intermediate (**C**, triangle) and terminal villi (**D**, triangle).

**FIGURE 3:**
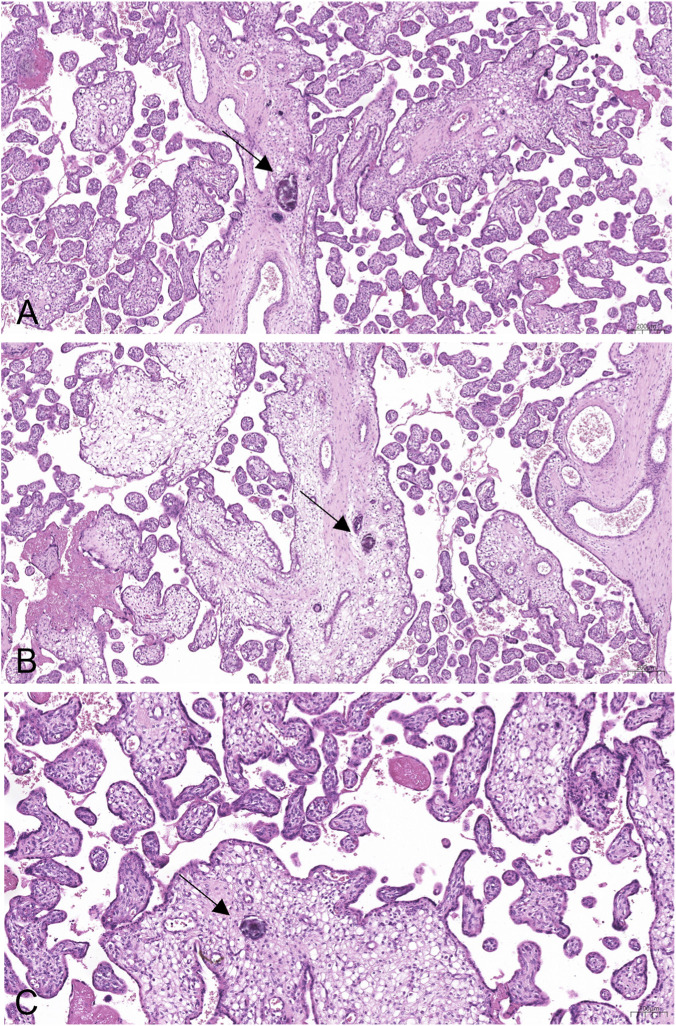
Sections of placental parenchyma, stained with hematoxylin and eosin (EE). Evident intravascular calcifications affecting the stem villi (**A**, **B**, arrow) and the intermediate villi (**C**, arrow).

Examination of the placental tissue revealed vacuolisation and oedema of the chorionic villi, accompanied by the accumulation of calcific deposits within vascular structures (Figures 2, 3).

**FIGURE 4:**
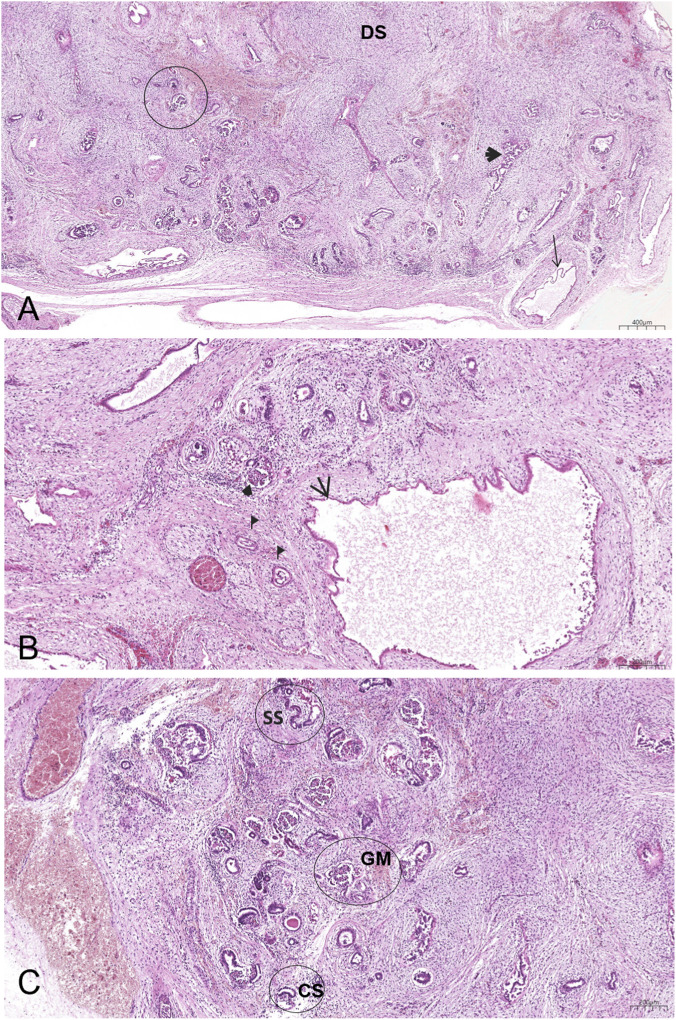
Histopathological picture of renal dysplasia, stained with hematoxylin and eosin (EE). **(A)** Dysplastic tubules (arrowhead) surrounded by dysplastic stroma (DS), dysplastic cysts (arrow) and irregular glomerules (circle). **(B)** Wall of dysplastic cyst (arrows), irregular glomeruli (arrowhead), tubules surrounded by fibromuscular collars (flags). **(C)** Different stages of glomerular maturation ranging from immature C-shaped (CS) and S-shaped (SS) bodies to mature forms (GM) with irregularities.

Renal tissue exhibited features consistent with congenital renal dysplasia, a developmental anomaly characterised by aberrant nephrogenesis, cystic change, and disorganised parenchymal architecture (Figure 4). Renal dysplasia is defined by microscopic criteria rather than by macroscopic examination of the whole organ; described diagnostic features include primitive tubules with fibrous or muscular mesenchymal collars, metaplastic elements (typically cartilage), disorganised parenchymal architecture, and aberrant nephron differentiation (Hinchliffe et al., 1991; Woolf et al., 2004). Although the available material consisted of tissue samples rather than whole kidneys, the examined sections were sufficient to apply these microscopic criteria and support the histological diagnosis. However, partial sampling precludes determination of the extent and distribution of the lesion (unilateral, bilateral, segmental) and does not allow definitive classification of entities requiring whole-organ macroscopic or quantitative assessment, such as multicystic dysplastic kidney or renal hypoplasia.

The diagnosis of SCT was histologically confirmed, demonstrating the presence of tissues derived from all three germ layers, consistent with its classification as a congenital germ cell tumour.

For toxicological evaluation, biological specimens consisting of FFPE tissues were submitted to the Advanced Translational Diagnostics Laboratory, where they were stored at ambient temperature pending sample preparation. Tissues selected for heavy metal analysis—based on their known relevance in toxicological bioaccumulation—comprised placental tissue, hepatic parenchyma, and renal tissue. Initially, FFPE samples were processed to remove paraffin using a standardized protocol (Di Candia et al., 2022). Tissue blocks were placed in a paraffin dispenser to initiate paraffin dissolution, followed by sequential incubations in xylene and graded ethanol solutions of increasing purity to ensure complete deparaffinisation and rehydration of the samples. As a contamination control, paraffin was sampled from the same FFPE block(s) after carefully removing and cleaning the tissue, and this block-matched paraffin was processed and analysed in parallel with the corresponding specimens to assess any contribution of metals from the embedding medium. Following sample preparation, the tissues underwent quantitative elemental analysis using inductively coupled plasma–mass spectrometry (ICP-MS) for the determination of heavy metal content (Gélinas et al., 1997).

Target elements were selected *a priori* as part of a predefined multi-element analytical panel to capture both essential elements (Co, Cr, Cu, Mn, Ni, Se, Zn) and non-essential/toxic metals (Al, Cd, Hg, Pb, Sb) with relevance to prenatal exposure and reported placental transfer, and to enable comparison with the biomonitoring and epidemiological literature (Esteban-Vasallo et al., 2012; Jagodić et al., 2022; Zhang et al., 2023). Importantly, element selection was not based on a facility-specific list of chemicals used in textile processing. The company’s occupational risk-assessment documentation/chemical inventory was not available to the authors; therefore, the analytical approach could not be restricted to substances formally documented as present at the workplace. Metal concentrations were quantified as total elemental content; no chemical speciation was performed. Therefore, the analysis cannot distinguish between different oxidation states/chemical forms or between bioavailable and poorly bioavailable fractions.

Each tissue sample was analysed in triplicate, and results are reported as the mean of the three measurements for each specimen. Two distinct placental samples were processed and analysed; for the placenta, the two-sample means were compared, and the higher mean was retained for conservative reporting. The results revealed markedly elevated concentrations of several toxic metals across all examined tissues. Specifically, the placental tissue exhibited chromium (Cr) (327.93 ppb), high levels of nickel (Ni) (182.27 ppb), and detectable concentrations of mercury (Hg) (11.77 ppb) and lead (Pb) (41.72 ppb). In the hepatic parenchyma, the concentrations were notably higher, with Cr at 806.22 ppb, Ni at 349.64 ppb, Hg at 90.67 ppb, and Pb at 338.97 ppb. Similarly, the renal tissue demonstrated Cr at 222.10 ppb, Ni at 109.59 ppb, Hg at 34.59 ppb, and Pb at 46.91 ppb. Above-normal concentrations of aluminum in the liver and copper in the kidney were also detected; however, these findings are not considered uniquely relevant to the assessment of occupational exposure. These results are summarised in Table 1 together with the reference (“background”) concentrations from unexposed cohorts to facilitate direct visual comparison.

The occupational medicine assessment focused on reconstructing the patient’s professional activity, particularly in relation to the textile production cycle, which encompasses multiple stages—such as fabric preparation and washing, spinning, weaving, dyeing, and finishing—each of which involves the potential use of a wide array of chemical substances. We conducted a retrospective, task-based occupational exposure interview to qualitatively characterise the patient’s work processes and potential exposure pathways. The patient reported employment in the textile sector from 2002 to 2021, working 8 h/day from Monday to Friday with intermittent Saturday shifts (6–8 h), sometimes sustained for up to approximately six consecutive months in certain years. Her duties spanned weaving (2002–2003), textile sample preparation (2003–2007), and mainly quality control at inspection machines (2007–2021), during which she had brief, periodic laboratory-based removal from exposure. During weaving, she worked on untreated (raw) textiles in a dedicated weaving area and described frequent contact with greasy materials, with visible residues on hands and clothing and an acrid odour. From 2007 onward, her main activity involved inspection immediately downstream of dyeing/finishing in adjacent halls, handling treated textiles described as dusty and occasionally sticky, again leaving residues on skin and workwear. At the inspection stations, manual fabric handling accounted for most of the shift (approximately 80%), with machine cleaning at each roll change (approximately 20%) and routine use of compressed air for cleaning in the presence of airborne fibres/dust. She reported recurrent irritant dermatitis affecting hands, arms, and legs (less frequently the face), and episodic mucosal/respiratory irritation (eye/nasal burning, cough, headache), with symptom fluctuation during periods of removal from exposure. Cleaning of equipment was typically performed in the production area using alcohol-based products supplied in bottles (approximately one bottle per week) applied to soaked cloths (including fabric scraps), without welding or metalworking; she noted intermittent fine “powder” formation when machine cylinders malfunctioned. Personal protective equipment was reportedly limited to safety shoes, with no routine use of gloves or respiratory protection; cotton workwear was laundered at home at the worker’s discretion, handwashing was limited (approximately twice per day) without barrier creams, and eating/drinking often occurred at the workstation. Work areas were enclosed, lacked local exhaust ventilation, and relied on minimal natural ventilation, with occasional fans in summer. The patient was not aware of any routine industrial hygiene monitoring (e.g., air or dust sampling) and did not recall receiving safety data sheets; occupational health surveillance by the company physician reportedly occurred but was sometimes delayed.

Overall, this qualitative reconstruction supports sustained opportunities for dermal contact and inhalation of fibres/dust/aerosols in proximity to treated textiles, while acknowledging uncertainty inherent to retrospective exposure assessment in the absence of employment records or quantitative measurements.

Textile processing environments may involve complex mixtures of chemical agents, including formaldehyde and other volatile compounds, plasticisers and finishing-related additives (e.g., phthalates, alkylphenol ethoxylates), polycyclic aromatic hydrocarbons, aromatic amines potentially released from azo dyes, organotin compounds, and heavy metals (including Hg, Cd, Pb, Ni, and hexavalent Cr) (Malik and Bharti, 2010; Zeiner et al., 2007; Sarwar and Khan, 2022; Khan and Malik, 2014).

In light of the integrated toxicological findings—particularly the elevated concentrations of Cr, Ni, Hg, and Pb in the placenta and fetal tissues—as well as the results of serial allergological patch tests conducted on the patient, there is substantiated evidence of occupational exposure to multiple hazardous agents, including heavy metals and formaldehyde. Although the patient was not directly involved in the dyeing or finishing stages themselves, her role in fabric quality control required direct handling of textiles immediately following these processes, implying significant indirect exposure to residual chemical agents present on the materials. Moreover, her responsibilities included the manual cleaning of machinery, a task that likely involved contact with processing residues enriched with potentially toxic substances.

Ultimately, the integration of anatomopathological, toxicological, and occupational medicine investigations provided converging evidence consistent with an occupationally mediated contributory role in the observed fetal phenotype, supporting biological plausibility within the limits of a single-case design.

## 4 Diagnostics

**TABLE 1:** Heavy metal concentrations from toxicological analyses are shown, with values above the normal range highlighted in red. Mean ± SD values are expressed on a dry weight basis where applicable. Chromium reference ranges were derived from adults, while mercury values are reported as mean (25th–75th percentiles).

Heavy metal	Placenta		Liver		Kidney	
	Case	Literature (mean ± SD)	Case	Literature (mean ± SD)	Case	Literature (mean ± SD)
Al (ppm)	3.45	4.82 ± 5.73 (Cerrillos et al., 2019)	9.65	2.69 ± 0.89 (Gélinas et al., 1997)	5.19	17.35 ± 4.69 (Gélinas et al., 1997)
		4.53 ± 0.81 (Roverso et al., 2019)				
Cr (ppb)	**327.93**	83.24 ± 28.10 (Cerrillos et al., 2019)	**806.22**	83 ± 28 (Vuoti et al., 2022)	222.10	2.9–298 (adult) (Dudek-Adamska et al., 2018)
		38 ± 23 (Roverso et al., 2019)				
Mn (ppm)	0.10	1.66 ± 9.56 (Cerrillos et al., 2019)	1.34	7.14 ± 1.09 (Gélinas et al., 1997)	0.45	2.02 ± 0.25 (Gélinas et al., 1997)
		0.57 ± 0.17 (Roverso et al., 2019)				
Co (ppb)	2.74	6 ± 4 (Iyengar and Rapp, 2001a)	13.66	50 ± 23 (Gélinas et al., 1997)	5.95	62 ± 23 (Gélinas et al., 1997)
		29 ± 34 (Roverso et al., 2019)				
Ni (ppb)	**182.27**	39.27 ± 23.33 (Cerrillos et al., 2019)	349.64	522 ± 151 (Gélinas et al., 1997)	109.59	518 ± 110 (Gélinas et al., 1997)
		113 ± 30 (Roverso et al., 2019)				
Cu (ppm)	0.83	0.97 ± 0.24 (Cerrillos et al., 2019)	45.23	234.02 ± 48.17 (Gélinas et al., 1997)	16.50	6.99 ± 1.45 (Gélinas et al., 1997)
		5 ± 1.2 (Roverso et al., 2019)				
Zn (ppm)	5.36	8.43 ± 0.97 (Cerrillos et al., 2019)	130.49	979.5 ± 111.9 (Gélinas et al., 1997)	62.20	103.8 ± 13.3 (Gélinas et al., 1997)
		58.8 ± 8.3 (Roverso et al., 2019)				
As (ppb)	1.33	1.2–19.6 (Vuoti et al., 2022)	2.38	—	1.11	—
Se (ppb)	129.71	190± −41 (Iyengar and Rapp, 2001a)	464.01	—	138.75	—
		130 ± 13 (Roverso et al., 2019)				
Cd (ppb)	3.16	17.24 ± 12.20 (Cerrillos et al., 2019)	2.89	64 ± 44 (Gélinas et al., 1997)	1.61	72 ± 38 (Gélinas et al., 1997)
		44 ± 21 (Roverso et al., 2019)				
Sb (ppb)	1.83	12 ± 3 (Roverso et al., 2019)	3.90	19 ± 10 (Gélinas et al., 1997)	1.06	47 ± 16 (Gélinas et al., 1997)
Hg (ppb)	**11.77**	6 ± 3 (Roverso et al., 2019; Tong et al., 2021)	**90.67**	21.7 (12.4–31.7) (Tong et al., 2021)	**34.59**	11.4 (7.11–15.0) (Tong et al., 2021)
		9.1 ± 3 (Vuoti et al., 2022), 7.29 ± 9.1 (Grant et al., 2010)				
Pb (ppb)	41.72	41.99 ± 14.80 (Cerrillos et al., 2019)	**338.97**	243 ± 92 (Gélinas et al., 1997)	46.91	137 ± 49 (Gélinas et al., 1997)
		50 ± 62 (Roverso et al., 2019)				

## 5 Patient perspective

“I worked in the textile industry for over 20 years and experienced recurrent skin and mucosal symptoms during my employment. The pregnancy outcome had a profound emotional impact, culminating in termination due to severe fetal malformations. In the subsequent reassessment, occupational exposures were considered a likely contributory factor. I hope this report supports better prevention, timely risk recognition, and protection for pregnant workers.”

## 6 Discussion

Worker protection and health are a critical public health concern, particularly when occupational exposure intersects with vulnerable life stages such as pregnancy (Rovira and Domingo, 2019; Akhtar et al., 2018). In this case, gaps in risk recognition, exposure assessment, and protective measures limited prevention and delayed integration of relevant aetiological indicators into the initial diagnostic hypothesis; ultimately, a diagnosis was achieved through multidisciplinary collaboration.

During pregnancy, standard follow-up protocols were appropriately implemented (National Institute for Health and Care Excellence (NICE), 2021; World Health Organization, 2016), including routine ultrasound imaging, screening for teratogenic infections, and karyotypic analysis. Maternal serology showed Rubella IgG positivity with IgM negativity and CMV IgG positivity with IgM negativity, consistent with pre-existing immunity/prior exposure and without serological evidence of recent primary infection (Centers for Disease Control and Prevention [CDC], 2024; American College of Obstetricians and Gynecologists [ACOG], n.d.); the infectious evaluation and karyotype were unremarkable.

Nevertheless, the pregnancy was electively terminated due to the presence of a severe malformative condition. Subsequent autopsy revealed, in addition to a sacrococcygeal teratoma, the presence of phocomelia affecting one upper limb—a malformation widely recognized in the scientific literature as potentially linked to exogenous toxic or pharmacological exposure (Lee et al., 2021; Bermejo-Sánchez et al., 2011). The initial histopathological examination reported no significant parenchymal alterations, and contemporaneous toxicological testing was not performed. In light of the postmortem findings, an expanded aetiological work-up including toxicology could have provided clinically relevant information, but measurements were not available.

Years later, a decision was made to re-examine the case based solely on the available clinical and instrumental documentation, along with FFPE tissues. Upon slide review and preparation of new histological sections, structural abnormalities were identified that were suggestive of changes reported in contexts of heavy-metal exposure/accumulation. While the observed morphological changes were non-specific, they raised the suspicion of compromised placental function. One plausible etiological hypothesis is chronic *in utero* exposure to heavy metals, which are well-documented to interfere with placental development—particularly villous maturation and vascular integrity (Gundacker and Hengstschläger, 2012; Reichrtová et al., 1998). Recent transcriptomic data further suggest that placental metal burden may be associated with altered gene-expression patterns involving cellular stress and signaling pathways. Although these findings do not establish causality in the present case, they provide additional mechanistic support for interpreting the placental abnormalities and adverse fetal outcome observed here (Kuzukiran et al., 2024). Heavy metals possess the ability to cross the placental barrier and accumulate within fetal tissues, including renal structures (Soria-Contreras et al., 2022; Casey and Robinson, 1978; Suzuki et al., 1979; Lutz et al., 1996; Jagodić et al., 2022).

Their nephrotoxic effects are mediated through diverse mechanisms, such as oxidative stress, mitochondrial dysfunction, inflammation, and epigenetic dysregulation—all of which may contribute to aberrant renal morphogenesis (Lee et al., 2015; Hernandez et al., 2013). Given their non-specificity, these findings required confirmation through targeted toxicological testing.

Toxicological analysis in the present case was constrained by the unavailability of fresh or frozen tissue; therefore, we applied a dedicated analytical workflow to extract and quantify heavy metals from formalin-fixed, paraffin-embedded (FFPE) tissues, while explicitly accounting for potential exogenous contamination from histological processing reagents (Di Candia et al., 2022). Under these constraints, only heavy metals (and not formaldehyde) could be investigated with acceptable analytical reliability. We recognise that FFPE entails intrinsic pre-analytical limitations: formalin fixation can promote element-specific leaching and redistribution, and fixation-related protein cross-linking may reduce access to metal-binding sites, potentially underestimating true tissue burdens (Koizumi et al., 1994; Shiurba et al., 1998; Bogen et al., 2009). Additional bias may arise during dehydration, clearing, and embedding, including partial masking of selected elements and reagent-derived contamination, particularly for ubiquitous metals in laboratory matrices (Johnston et al., 2009; Lee et al., 1995; McCormack et al., 2020). To mitigate this risk, we separately analysed the embedding paraffin; moreover, if substantial reagent contamination had occurred, concentrations would be expected to homogenise across samples. Instead, we observed markedly elevated yet clearly heterogeneous metal levels across organs, arguing against a uniform exogenous source and supporting a genuine tissue signal. Notably, nickel and chromium have been reported to show negligible leaching and to remain reliably quantifiable in FFPE material when optimised ICP-MS workflows are applied (Gellein et al., 2008). Accordingly, while partial loss and low-level contamination cannot be entirely excluded, the magnitude and organ-specific distribution of the detected metals support interpretation of the measured burdens as realistic and best considered conservative estimates of *in vivo* exposure.

As noted in the Methods, the facility’s chemical inventory/occupational risk-assessment documentation was unavailable; exposure reconstruction therefore relied on the task-based occupational history and clinical context. The most plausible exposure pathways were dermal contact with treated textiles and inhalation of fibres/dust/aerosols in poorly ventilated enclosed work areas, in the absence of gloves or respiratory protection.

Although metals and metalloids are ubiquitous and can accumulate in the maternal body through the food chain and other exposure routes, prenatal exposure may adversely affect the fetus—particularly during early development, including neurodevelopment (Meyrueix et al., 2019). Background exposure is higher in urban and industrialised settings for several elements (e.g., Mn, Zn, Cr, Cu, Ni); however, even low gestational exposure to As, Pb, Cd, and Hg warrants attention given potential fetal toxicity. Placental handling differs by element: Cd may preferentially accumulate in placental tissue, whereas As, Cr, Cu, Pb, Mn, Ni, Se, and Zn can be transferred from maternal tissues to the placenta and subsequently to the fetus (Serafim et al., 2012).

Given that thresholds for fetal heavy-metal exposure and toxicity remain incompletely defined, we interpreted tissue concentrations against ranges reported in unexposed reference populations as contextual benchmarks rather than definitive individual-level thresholds. This approach accounts for substantial inter-individual and population-dependent variability driven by environmental background, diet, smoking status, geography, analytical matrices, and gestational timing (Grant et al., 2010; Esteban-Vasallo et al., 2012; Bocca et al., 2019). In the absence of Italian reference data for heavy-metal concentrations in fetal liver and kidney, comparisons were prioritised to Italian placental cohorts when feasible; otherwise, fetal organ values were benchmarked against the most methodologically comparable fetal/perinatal tissue datasets available, acknowledging imperfect matching for urbanicity and socioeconomic context.

Accordingly, we focused on trace elements of occupational/toxicological relevance (Co, Cr, Cu, Mn, Ni, Se, Zn; Al, Cd, Hg, Pb, Sb) and did not quantify major macro-elements (Ca, Fe, K, Mg, Na, P).

Placental tissues presented non-anomalous levels of essential metals involved in iron-channel transport, such as Mn, Cu, Co, and Zn, in line with previous evidence (Cerrillos et al., 2019; Roverso et al., 2019; Iyengar and Rapp, 2001b). Conversely, in the fetal liver, Mn, Cu, and Zn concentrations were substantially higher than placental levels, supporting the notion that the liver may act as a major storage site for these essential elements during fetal life.

Moreover, Pb is also generally higher in the liver than the concentration found in other non-exposed fetal organs, since, as iron, it easily crosses the placental barrier thanks to its high affinity for fetal hemoglobin. Compared to literature averages, this metal is higher (338.97 ppb) than normal (average 243 ppb), while it is normal at the placental level (Gélinas et al., 1997).

Notably, Ni levels in the analysed placental tissues were higher (182.27 ppb) than the mean values reported in two recent studies from an Italian cohort (113 ppb) and a Spanish cohort (39.27 ppb) (Cerrillos et al., 2019; Roverso et al., 2019). Even when considering a Portuguese cohort reporting mean placental values of 65 ± 47 ppb (Serafim et al., 2012), the Ni concentration observed in our placental samples was at least approximately two-fold higher. Previous evidence has linked occupational Ni exposure to increased risk of pregnancy loss, plausibly mediated by adverse effects on placental structure and function (Reichrtová et al., 1998).

Of concern were the Hg concentrations measured across all analysed samples. Total Hg concentrations, which were not speciated into inorganic and organic forms, fall within ranges reported in association with neural tube defects. Notably, Hg levels in liver and kidney tissues were 3–4 times higher (placenta 11.77 ppb; liver 90.67 ppb; kidney 34.59 ppb) than those reported in a reference group with neural tube defects (average placenta 6 ppb; liver 21.7 ppb; kidney 11.4 ppb) (Roverso et al., 2019; Tong et al., 2021). Another study reported mean placental Hg concentrations of 9.1 ppb (Vuoti et al., 2022).

Furthermore, elevated total Cr values were detected. Placental concentrations were four-to ten-fold higher (327.93 ppb) than mean values reported in reference studies (Italian cohort average: 38 ppb; Spanish cohort average: 83.24 ppb). In fetal liver, Cr concentrations were approximately ten-fold higher (806.22 ppb) than previously reported literature averages (83 ppb) (Gundacker and Hengstschläger, 2012). However, no population reference data are available for Cr levels in fetal kidney tissue; adult reference intervals (2.9–298 ppb) (Dudek-Adamska et al., 2018) are not directly applicable to fetal tissues, and the kidney Cr concentration cannot therefore be reliably benchmarked.

Importantly, the detection of total metal concentrations in tissues should be interpreted as evidence of exposure and tissue burden, not as a direct proxy for the toxicologically active (bioavailable) fraction or effective internal dose. Because our digestion-based analysis quantified total elemental content without chemical speciation, we cannot distinguish more bioavailable from less bioavailable forms; therefore, any biological interpretation should be made prudently and integrated with exposure history and the evidence base (Templeton et al., 2000; UK Health Security Agency, 2022; EFSA Panel on Contaminants in the Food Chain (CONTAM), 2014).

To contextualise the toxicological findings and assess the plausibility of embryotoxic/teratogenic effects, we conducted a targeted literature search across Scopus, PubMed, and Web of Science on prenatal exposure to chromium, nickel, mercury, and lead; placental transfer and biomonitoring matrices (maternal blood, placenta, and umbilical cord blood); and congenital anomalies/birth defects, with particular attention to renal and urogenital outcomes. Evidence was prioritised hierarchically, giving precedence to systematic reviews and meta-analyses and, when primary studies were required, to temporality-based analytical designs (prospective cohorts and case-control studies). The resulting evidence base was then integrated within the occupational medicine assessment to interpret the confirmed heavy-metal findings against the epidemiological and toxicological literature (Esteban-Vasallo et al., 2012; Li et al., 2022; Eaves et al., 2023; Ou et al., 2025). Study quality was appraised qualitatively by considering exposure assessment, outcome ascertainment, confounder control, and the risk-of-bias approaches reported by the original authors; cross-sectional biomonitoring studies were used to characterise exposure patterns and materno–fetal transfer.

Overall, the human literature on prenatal metal exposure remains heterogeneous and predominantly observational and is therefore best interpreted as supporting associations rather than definitive individual-level causal attribution. Within this framework, the most informative evidence concerned congenital outcomes for which synthesis and temporality-based designs are available, including orofacial clefts, congenital heart defects, chromosomal abnormalities, and congenital anomalies of the kidney and urinary tract (Ni et al., 2018; Wang et al., 2022; Iwaya et al., 2023; Liu et al., 2023). Biomonitoring studies were used to contextualise exposure patterns and placental transfer in comparable pregnancy settings (Li et al., 2019; Yüksel et al., 2021; Jagodić et al., 2022; Zhang et al., 2023), while mechanistic placental evidence was considered to support biological plausibility where relevant, including experimental data on chromium transport in perfused human placental tissue and recent transcriptomic evidence related to placental metal burden (Al-Sannan et al., 2019; Kuzukiran et al., 2024).

In parallel, the occupational history enabled verification of plausible workplace contact with the identified agents, including formaldehyde, for which teratogenic potential has been described in the literature (Thrasher and Kilburn, 2001; Duong et al., 2011; Xu et al., 2017; Al-Kalaa et al., 2020).

Congenital anomalies may also arise from sporadic or *de novo* genetic events occurring in germ cells or during embryogenesis (Wojcik and Agrawal, 2020), and such mechanisms cannot be excluded at the individual level. Nevertheless, the present case is characterised by a particularly strong and convergent exposure profile: the occupational history indicates sustained workplace contact with formaldehyde, corroborated by clinically documented cutaneous sensitisation, and toxicological analyses revealed markedly elevated fetal chromium concentrations. Taken together, these elements provide a robust exposure signal and strengthen biological plausibility for an environmentally mediated contribution. Accordingly, while alternative developmental mechanisms remain possible, the occupational exposure pathway should be regarded as a likely contributory factor and interpreted as central to the overall aetiological appraisal.

Of note, the patient discontinued factory work at approximately 5 weeks’ gestation. Two non-mutually exclusive mechanisms may be considered when interpreting the temporal relationship between maternal workplace departure and the observed fetal phenotype. First, even short-duration exposures occurring in the peri-conceptional and early embryonic period may have disproportionate impact, because the highest susceptibility to major structural malformations broadly aligns with early embryogenesis and organogenesis (Brent, 2004; Sadler, 2017). Second, for several metals, long-term occupational exposure can generate an endogenous maternal reservoir; pregnancy-related physiological changes (including increased bone turnover) can mobilise stored metals and sustain fetal exposure even after external exposure ceases, particularly for lead stored in the skeleton and transferred to the fetus during gestation (Gulson et al., 1997; Osorio-Yáñez et al., 2021; Ali Daoud et al., 2023). In addition, other metals show slow toxicokinetics and tissue persistence (e.g., cadmium with multi-year to multi-decade half-lives, and chromium with slow elimination after occupational exposure), and mercury forms can cross the placenta with relatively slow elimination, supporting the plausibility of residual internal burden contributing to fetal exposure dynamics (Petersen et al., 2000; Nordberg et al., 2018; Dack et al., 2021). Given the lack of contemporaneous biomonitoring and quantitative exposure reconstruction, the relative contribution of early gestational exposure versus mobilisation of pre-existing maternal body burden cannot be determined; accordingly, these considerations are presented only as a mechanistic framework for interpreting the case.

An additional and clinically relevant finding in this case was the presence of a sacrococcygeal teratoma (SCT), a rare congenital tumour arising from pluripotent cells in the caudal embryonic axis. Although SCT is generally framed as a disorder of early embryogenesis and primordial germ cell development, its precise aetiology remains incompletely defined (Phi, 2021). At present, there is no conclusive epidemiological evidence establishing a direct causal relationship between SCT and parental occupational exposures; however, the broader literature on prenatal exposures and paediatric germ cell tumours supports the biological plausibility of investigating this hypothesis. Specifically, prenatal exposure to air toxics and related volatile chemicals has been examined in relation to malignant germ cell tumours in early childhood (Hall et al., 2019), and population-based studies have explored associations between parental occupation and childhood germ cell tumours (Hall et al., 2021). In parallel, maternal occupational exposure to dichloromethane during pregnancy has been proposed as a potential risk factor for selected fetal tumours, although available data remain limited (Spinder et al., 2019; Hall et al., 2021). Within mechanistic and reproductive-toxicology frameworks, polycyclic aromatic hydrocarbons and phthalates—two exposure classes relevant to occupational and environmental settings—have been linked to placental dysfunction, endocrine disruption, and molecular perturbations during pregnancy (Dai et al., 2023; Warner et al., 2021), while formaldehyde has demonstrated teratogenic potential in experimental models (Al-Kalaa et al., 2020). In the present case, SCT co-occurred with multiple severe fetal anomalies and placental alterations in a context of possible maternal occupational exposure to toxicants, and alongside markedly elevated yet tissue-specific metal burdens in the analysed specimens; accordingly, the observation is best interpreted as hypothesis-generating with respect to a potential contribution of environmental/occupational teratogens to SCT pathogenesis, while recognising that individual exposure magnitude and source attribution cannot be determined. While causality cannot be inferred from a single case, this finding supports the need for systematic case aggregation and more granular exposure assessment in future studies to evaluate whether SCT risk may be modified by complex maternal exposure mixtures.

## 7 Conclusion

Occupational health surveillance is essential, particularly for pregnant workers, given the potential impact of toxic exposures on fetal development. In cases of fetal malformations, the aetiological assessment should include a systematic appraisal of potential environmental and occupational contributors, integrating task-based exposure history and, where clinically justified, toxicological testing. Retrospective toxicological investigation may remain informative even years later when paraffin-embedded tissue is available, provided that validated analytical workflows and appropriate contamination controls are applied. While sacrococcygeal teratoma (SCT) is typically framed as a disorder of early embryogenesis, the present observation is best regarded as hypothesis-generating and supports further epidemiological and mechanistic research on whether complex maternal exposure mixtures may modify SCT risk. Inadequate control of workplace hazards can have substantial health, organizational, and compensation-related implications, underscoring the need for effective preventive measures for pregnant workers. For individuals planning pregnancy in high-risk occupational settings, preconception counselling and precautionary work modification or temporary reassignment, guided by occupational risk assessment, may be considered.

## Data Availability

The original contributions presented in the study are included in the article/Supplementary Material, further inquiries can be directed to the corresponding author.
