# Supplementary material for: Heavy metals detected in fetal and placental tissues in a pregnancy complicated by severe fetal growth impairment and sacrococcygeal teratoma: a case report
Source: Front Toxicol. 2026 Mar 19;8:1722241. doi: 10.3389/ftox.2026.1722241 (PMC13044029; doi:10.3389/ftox.2026.1722241)
Supplement: Supplementary file 1 [file Table1.docx]

**Supplementary Material**

Supplementary Tables S1-S5

**Table S1. Heavy metal content in analytical blanks, reagent blanks, and paraffin blanks.**

| **Sample** | **Al (ppm)** | **Cr (ppb)** | **Mn (ppm)** | **Co (ppb)** | **Ni (ppb)** | **Cu (ppm)** | **Zn (ppm)** | **As (ppb)** | **Se (ppb)** | **Cd (ppb)** | **Sb (ppb)** | **Hg (ppb)** | **Pb (ppb)** |
| --- | --- | --- | --- | --- | --- | --- | --- | --- | --- | --- | --- | --- | --- |
| Blank | 0.03 | 7.1 | 0.7 | 0.05 | 1.1 | 1.1 | 7.1 | 0.02 | 0.00 | 0.08 | 0.01 | 0.0 | 0.8 |
| Reagent blank | 0.08 | 1.5 | 0.9 | 0.04 | 2.3 | 9.8 | 14.8 | 0.01 | 0.09 | 0.05 | 0.02 | 0.4 | 0.8 |
| Paraffin blank | 0.3 | 4.1 | 1.7 | 0.05 | 2.0 | 20.4 | 27.9 | 0.06 | 0.00 | 0.20 | 0.10 | 0.2 | 4.01 |

**Table S2. Heavy metal content in placental sample A1 (inter-day precision).**

| **Element** | **Inter-day replicates (n)** | **Day 1** | **Day 2** | **Day 3** | **Mean** | **SD** | **CV (%)** |
| --- | --- | --- | --- | --- | --- | --- | --- |
| Al (ppm) | 3 | 1.93 | 2.59 | 2.61 | 2.38 | 0.32 | 13.29 |
| Cr* (ppb) | 3 | 269.9 | 314.5 | 319.6 | 301.33 | 22.32 | 7.41 |
| Mn (ppm) | 3 | 0.12 | 0.14 | 0.14 | 0.13 | 0.01 | 7.07 |
| Co (ppb) | 3 | 2.45 | 2.45 | 3.31 | 2.74 | 0.41 | 14.81 |
| Ni (ppb) | 3 | 172.0 | 186.8 | 188.0 | 182.27 | 7.28 | 3.99 |
| Cu (ppm) | 3 | 0.73 | 0.84 | 0.87 | 0.81 | 0.06 | 7.40 |
| Zn (ppm) | 3 | 6.15 | 6.01 | 6.93 | 6.36 | 0.40 | 6.36 |
| As (ppb) | 3 | 0.47 | 0.66 | 0.60 | 0.58 | 0.08 | 13.75 |
| Se (ppb) | 3 | 137.7 | 159.1 | 182.3 | 159.70 | 18.21 | 11.40 |
| Cd (ppb) | 3 | 2.11 | 2.36 | 2.58 | 2.35 | 0.19 | 8.17 |
| Sb (ppb) | 3 | 1.48 | 1.98 | 1.26 | 1.57 | 0.30 | 19.15 |
| Hg** (ppb) | 2 | — | 12.03 | 11.50 | 11.77 | 0.27 | 2.25 |
| Pb (ppb) | 3 | 24.94 | 25.51 | 30.74 | 27.06 | 2.61 | 9.64 |

* Value expressed as total chromium. ** Value expressed as total mercury.

**Table S3. Heavy metal content in placental sample A3 (inter-day precision).**

| **Element** | **Inter-day replicates (n)** | **Day 1** | **Day 2** | **Day 3** | **Mean** | **SD** | **CV (%)** |
| --- | --- | --- | --- | --- | --- | --- | --- |
| Al (ppm) | 3 | 3.51 | 3.19 | 3.66 | 3.45 | 0.20 | 5.68 |
| Cr* (ppb) | 3 | 413.6 | 263.4 | 306.8 | 327.93 | 63.11 | 19.25 |
| Mn (ppm) | 3 | 0.10 | 0.089 | 0.10 | 0.10 | 0.01 | 5.38 |
| Co (ppb) | 3 | 2.45 | 2.45 | 3.31 | 2.74 | 0.41 | 14.81 |
| Ni (ppb) | 3 | 105.76 | 92.73 | 93.55 | 97.35 | 5.96 | 6.12 |
| Cu (ppm) | 3 | 0.82 | 0.79 | 0.88 | 0.83 | 0.04 | 4.51 |
| Zn (ppm) | 3 | 5.58 | 5.25 | 5.25 | 5.36 | 0.16 | 2.90 |
| As (ppb) | 3 | 1.59 | 1.08 | 1.33 | 1.33 | 0.21 | 15.62 |
| Se (ppb) | 3 | 124.69 | 123.44 | 140.99 | 129.71 | 7.99 | 6.16 |
| Cd (ppb) | 3 | 3.10 | 3.10 | 3.27 | 3.16 | 0.08 | 2.54 |
| Sb (ppb) | 3 | 1.65 | 1.68 | 2.15 | 1.83 | 0.23 | 12.53 |
| Hg** (ppb) | 2 | — | 8.43 | 10.88 | 9.66 | 1.22 | 12.69 |
| Pb (ppb) | 3 | 42.15 | 42.80 | 40.22 | 41.72 | 1.10 | 2.63 |

* Value expressed as total chromium. ** Value expressed as total mercury.

**Table S4. Heavy metal content in fetal liver parenchyma sample B6 (inter-day precision).**

| **Element** | **Inter-day replicates (n)** | **Day 1** | **Day 2** | **Day 3** | **Mean** | **SD** | **CV (%)** |
| --- | --- | --- | --- | --- | --- | --- | --- |
| Al (ppm) | 3 | 9.33 | 9.21 | 10.42 | 9.65 | 0.54 | 5.64 |
| Cr* (ppb) | 3 | 816.29 | 764.16 | 838.20 | 806.22 | 31.05 | 3.85 |
| Mn (ppm) | 3 | 1.37 | 1.26 | 1.39 | 1.34 | 0.06 | 4.27 |
| Co (ppb) | 3 | 13.14 | 12.39 | 15.45 | 13.66 | 1.30 | 9.53 |
| Ni (ppb) | 3 | 372.38 | 323.53 | 353.02 | 349.64 | 20.09 | 5.74 |
| Cu (ppm) | 3 | 45.62 | 42.52 | 47.56 | 45.23 | 2.08 | 4.59 |
| Zn (ppm) | 3 | 140.23 | 112.76 | 138.47 | 130.49 | 12.56 | 9.62 |
| As (ppb) | 3 | 2.19 | 2.04 | 2.90 | 2.38 | 0.38 | 15.78 |
| Se (ppb) | 3 | 453.52 | 423.76 | 514.75 | 464.01 | 37.88 | 8.16 |
| Cd (ppb) | 3 | 2.95 | 2.59 | 3.14 | 2.89 | 0.23 | 7.88 |
| Sb (ppb) | 3 | 4.00 | 4.71 | 2.98 | 3.90 | 0.71 | 18.22 |
| Hg** (ppb) | 2 | — | 83.69 | 97.65 | 90.67 | 6.98 | 7.70 |
| Pb (ppb) | 3 | 357.62 | 292.71 | 366.59 | 338.97 | 32.92 | 9.71 |

* Value expressed as total chromium. ** Value expressed as total mercury.

**Table S5. Heavy metal content in fetal kidney sample B5 (inter-day precision).**

| **Element** | **Inter-day replicates (n)** | **Day 1** | **Day 2** | **Day 3** | **Mean** | **SD** | **CV (%)** |
| --- | --- | --- | --- | --- | --- | --- | --- |
| Al (ppm) | 3 | 4.55 | 5.32 | 5.70 | 5.19 | 0.48 | 9.22 |
| Cr* (ppb) | 3 | 195.7 | 227.7 | 242.9 | 222.10 | 19.67 | 8.86 |
| Mn (ppm) | 3 | 0.40 | 0.46 | 0.50 | 0.45 | 0.04 | 9.07 |
| Co (ppb) | 3 | 5.23 | 5.90 | 6.72 | 5.95 | 0.61 | 10.24 |
| Ni (ppb) | 3 | 107.95 | 110.51 | 110.31 | 109.59 | 1.16 | 1.06 |
| Cu (ppm) | 3 | 14.41 | 16.89 | 18.20 | 16.50 | 1.57 | 9.53 |
| Zn (ppm) | 3 | 58.24 | 58.67 | 69.69 | 62.20 | 5.30 | 8.52 |
| As (ppb) | 3 | 1.13 | 0.92 | 1.28 | 1.11 | 0.15 | 13.30 |
| Se (ppb) | 3 | 119.13 | 137.54 | 159.59 | 138.75 | 16.54 | 11.92 |
| Cd (ppb) | 3 | 1.38 | 1.59 | 1.85 | 1.61 | 0.19 | 11.97 |
| Sb (ppb) | 3 | 1.03 | 1.64 | 0.51 | 1.06 | 0.46 | 43.57 |
| Hg** (ppb) | 2 | — | 33.03 | 36.15 | 34.59 | 1.56 | 4.51 |
| Pb (ppb) | 3 | 42.72 | 43.13 | 54.87 | 46.91 | 5.63 | 12.01 |

* Value expressed as total chromium. ** Value expressed as total mercury.
